# Endoscopic laser lithotripsy for a pancreaticojejunostomy stricture using a novel ultra-slim cholangiopancreatoscope via an endoscopic ultrasound-guided pancreatogastrostomy route

**DOI:** 10.1055/a-2771-4425

**Published:** 2026-01-22

**Authors:** Kimi Bessho, Takeshi Ogura, Junichi Nakamura, Nga Nguyen Trong, Hiroki Nishikawa

**Affiliations:** 1 13010 Pancreatobiliary Advanced Medical Center, Osaka Medical and Pharmaceutical University Hospital, Osaka, Japan; 2 2nd Department of Internal Medicine, Osaka Medical and Pharmaceutical University, Osaka, Japan; 3 Endoscopy Center, Osaka Medical and Pharmaceutical University Hospital, Osaka, Japan; 4 Department of Gastroenterology, Trong Nam Cancer Hospital, Hanoi, Vietnam

Endoscopic ultrasound-guided pancreaticogastrostomy (EUS-PD) is now attempted for failed endoscopic retrograde cholangiopancreatography (ERCP). After EUS-PD, antegrade procedures such as stone extraction and laser ablation can be tried. However, during antegrade procedures, a pancreatoscope must be inserted into the main pancreatic duct through an EUS-PD route. To insert the pancreatoscope, tract dilation techniques such as balloon dilation and metal stent deployment have been reported 1 2 . In cases of balloon dilation, however, the tract might rupture, and the cost of deploying a metal stent is high. To overcome this, an ultra-slim cholangiopancreatoscope with the unique characteristic of providing the working channel exit at the 3 o’clock position (7.8 Fr, Briview, SeeGen Co., Ltd, Shanghai, China) has become available. Technical tips for antegrade laser ablation of a pancreaticojejunostomy stricture (PJS) using this scope are described.

**Fig. 1:**
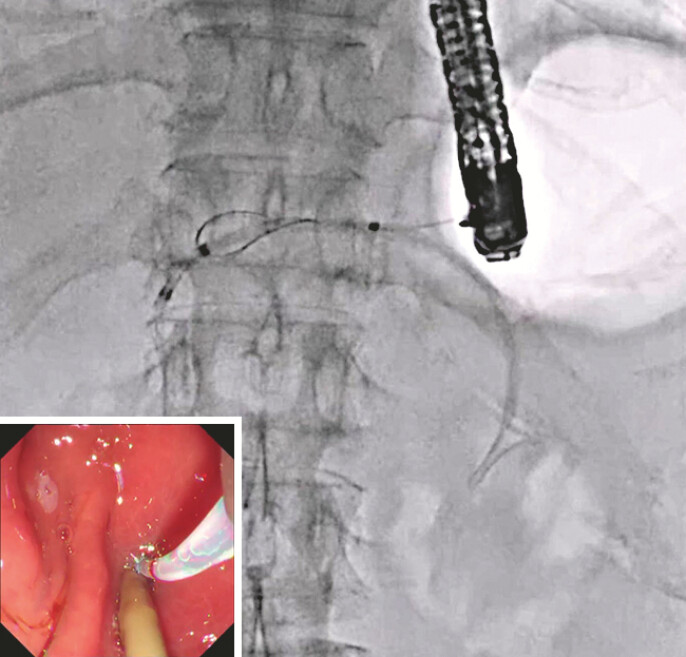
A 0.025-inch guidewire is deployed along the plastic stent.

**Fig. 2:**
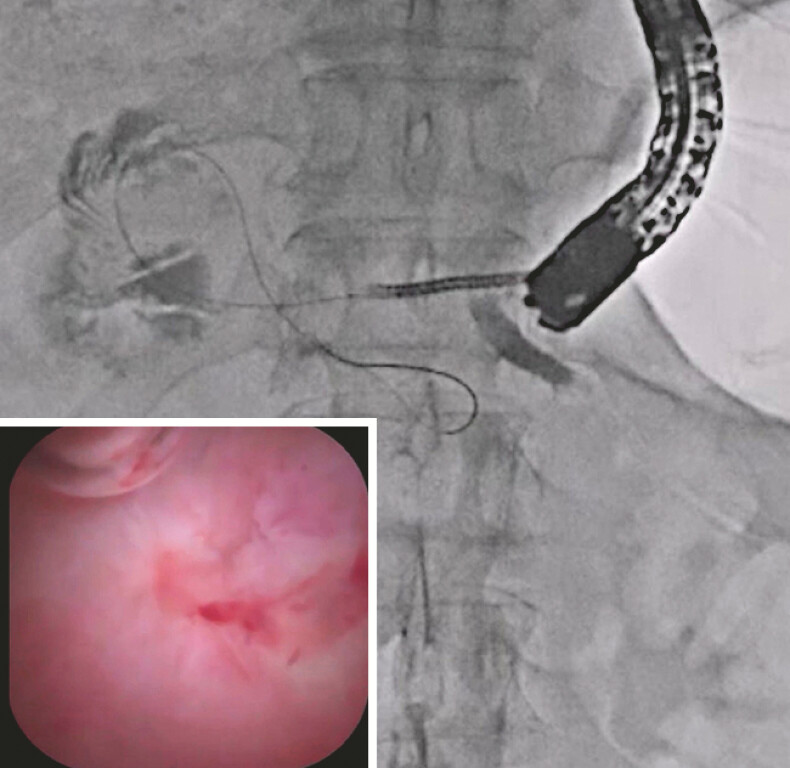
Novel pancreatoscope insertion is attempted without tract dilation, and it is successfully inserted into the main pancreatic duct.

**Fig. 3:**
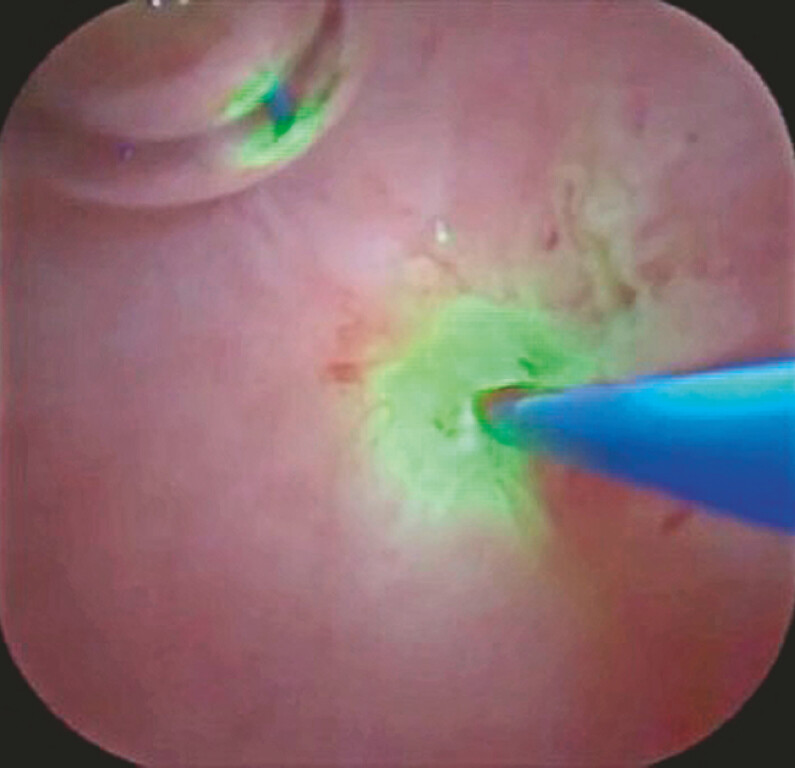
Laser ablation for the pancreaticojejunostomy stricture can be easily performed without mucosal injury.

**Fig. 4:**
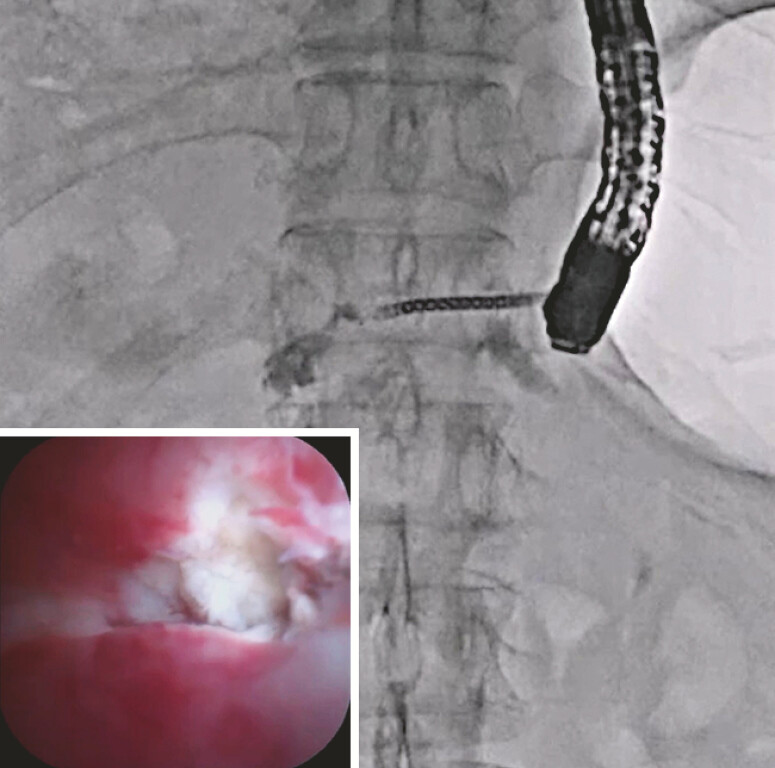
After antegrade laser ablation, stricture resolution is obtained.

**Fig. 5:**
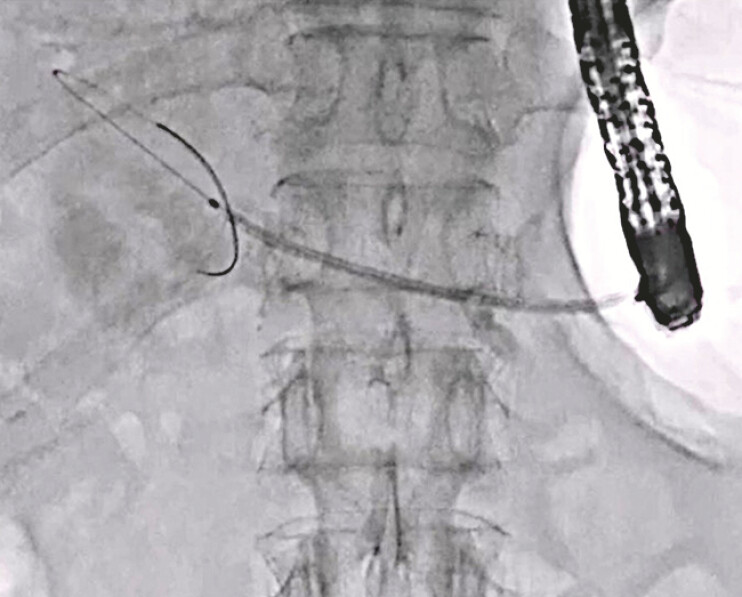
A plastic stent is deployed without any adverse events.

A 71-year-old man underwent pancreaticoduodenostomy due to pancreatic cancer 1 year earlier. He developed frequent pancreatitis due to a PJS and was therefore admitted to our hospital. First, he underwent EUS-PD using a 7-Fr plastic stent without any adverse events. Two weeks later, antegrade treatment was attempted for the PJS. First, a 0.025-inch guidewire was deployed along the plastic stent ( Fig. 1 ), and the stent was removed. Then, novel pancreatoscope insertion was attempted without tract dilation, and it was successfully inserted into the main pancreatic duct ( Fig. 2 ). The PJS was then identified. Because the probe was extracted from the 3 o’clock position, laser ablation for the PJS could be easily performed without mucosal injury ( Fig. 3 ). After antegrade laser ablation, stricture resolution was obtained ( Fig. 4 ). Finally, a plastic stent was deployed without any adverse events ( Fig. 5 , Video 1 ). This patient underwent stent removal after 3 months, with no stricture recurrence observed during the clinical follow-up.

Novel pancreatoscope insertion is attempted without tract dilation, and it is successfully inserted into the main pancreatic duct.Video 1

In conclusion, an antegrade procedure using this slim pancreatoscope may be useful because tract dilation is not needed.

Endoscopy_UCTN_Code_TTT_1AS_2AI
